# Down-regulation of MRPS23 inhibits rat breast cancer proliferation and metastasis

**DOI:** 10.18632/oncotarget.17888

**Published:** 2017-05-15

**Authors:** Yan Gao, Fuyan Li, Hong Zhou, Yi Yang, Ruimin Wu, Yijia Chen, Wei Li, Yang Li, Xueqin Xu, Changbin Ke, Zhijun Pei

**Affiliations:** ^1^ Department of PET Center and Institute of Anesthesiology and Pain, Taihe Hospital, Hubei University of medicine, Hubei, China

**Keywords:** MRPS23, breast cancer, metastasis, p53, p21 ^WAF1/CIP1^

## Abstract

Mitochondrial ribosomal protein S23 (MRPS23) has been shown to be involved in breast cancer cell proliferation and metastatic phenotypes of cervical cancer. Here we investigated its biological features in breast cancer for the first time. It demonstrated that knockdown of MRPS23 reduced breast cancer cell proliferation and induced apoptosis *in vitro*. Besides, shRNA targeting MRPS23 (shMRPS23) inhibited tumour proliferation and metastasis by blocking tumor angiogenesis in breast cancer xenograft rat model. Small animal positron emission tomography/computed tomography (PET/CT) with 2′-deoxy-2′-[^18^F] fluoro-D-glucose (FDG) was performed at four weeks after tumour cell injection. We found that FDG maximum standardized uptake value (SUVmax) significantly decreased by 31 ± 3% in the shMRPS23-treated group. But this change was not independent of metabolic tumour size. In addition, we also found that shMRPS23 could significantly suppress breast cancer metastasis through inhibiting epithelial mesenchymal transition (EMT) phenotype. The epithelial marker E-cadherin was increased, whereas the metastasis associated gene vimentin was decreased. Mechanistically, shMRPS23-treated tumours failed to progress through p53 and p21^WAF1/CIP1^ activation, but not cytochrome c-mediated pathway. These findings suggest that MRPS23 is a potential therapeutic target for interference of breast cancer proliferation, angiogenesis and metastasis.

## INTRODUCTION

Breast cancer is a major cause of cancer-related death, with about 1.2 million newly diagnosed cases every year and increasing numbers of cases in developing countries, including China [1, 2]. The reasons for high risk and mortality rate is that it can proliferate and metastasize in the lung, lymph node or elsewhere [3], especially in bone metastasis [4]. Although much effort is being spent on developing new therapeutic targets, the molecular mechanisms of breast cancer tumourigenesis and metastasis is still unclear, and the overall survival rate is still low as a consequence.

This study focused on a 22 kDa mitochondrial ribosomal protein S23 (MRPS23) that is located at 17q22–23. MRPS23 was reported to be overexpressed in various cancers, such as breast [5, 6], ovarian [7], colorectal [8], uterine leiomyoma [9] and hepatocellular carcinoma [10]. Furthermore, it has been known as a key genetic drivers that is essential for cell proliferation and is uniquely expressed in breast cancer patients [5]. MRPS23 is also more strongly associated with metastatic phenotypes in cervical cancer [11, 12]. These studies indicate that MRPS23 may exhibit a negative impact on proliferative and metastatic breast cancer. Nevertheless, the exact function of the molecule and the mechanism by which MRPS23 inhibits the proliferation and metastasis of breast cancer remain unknown.

Recent studies have examined the biological functions and the possible mechanisms of mitochondrial ribosomal proteins (MRPs) in cancers individually. Several evidence have shown that MRPs are involved in cell proliferation and mitochondrial apoptosis through the p21^WAF1/CIP1^ [13], p53 [14–16] and cytochrome c (Cyt c) -mediated pathway [17]. p21^WAF1/CIP1^ may be regulated through either p53-dependent [18] or p53-independent mechanism [19]. However, for the new target gene MRPS23, there are few reports as to whether this mechanism occurs. Angiogenesis, is a key process in pathological conditions that drives the progression of tumour [20]. In addition, epithelial mesenchymal transition (EMT) plays an important role in the metastatic process of breast cancer cells, of which the E-cadherin and vimentin proteins are key components [21]. Here, we investigated the expression and biological significance of MRPS23 in breast cancer, and demonstrated that shMRPS23 inhibits cell proliferation and survival by repressing p53 and p21^WAF1/CIP1^. We further described a critical role for MRPS23 as an important suppressor of proliferation and metastasis using ^18^F-FDG PET imaging.

## RESULTS

### Downregulation of MRPS23 inhibited breast cancer proliferation and induced apoptosis *in vitro*

**Figure 1:**
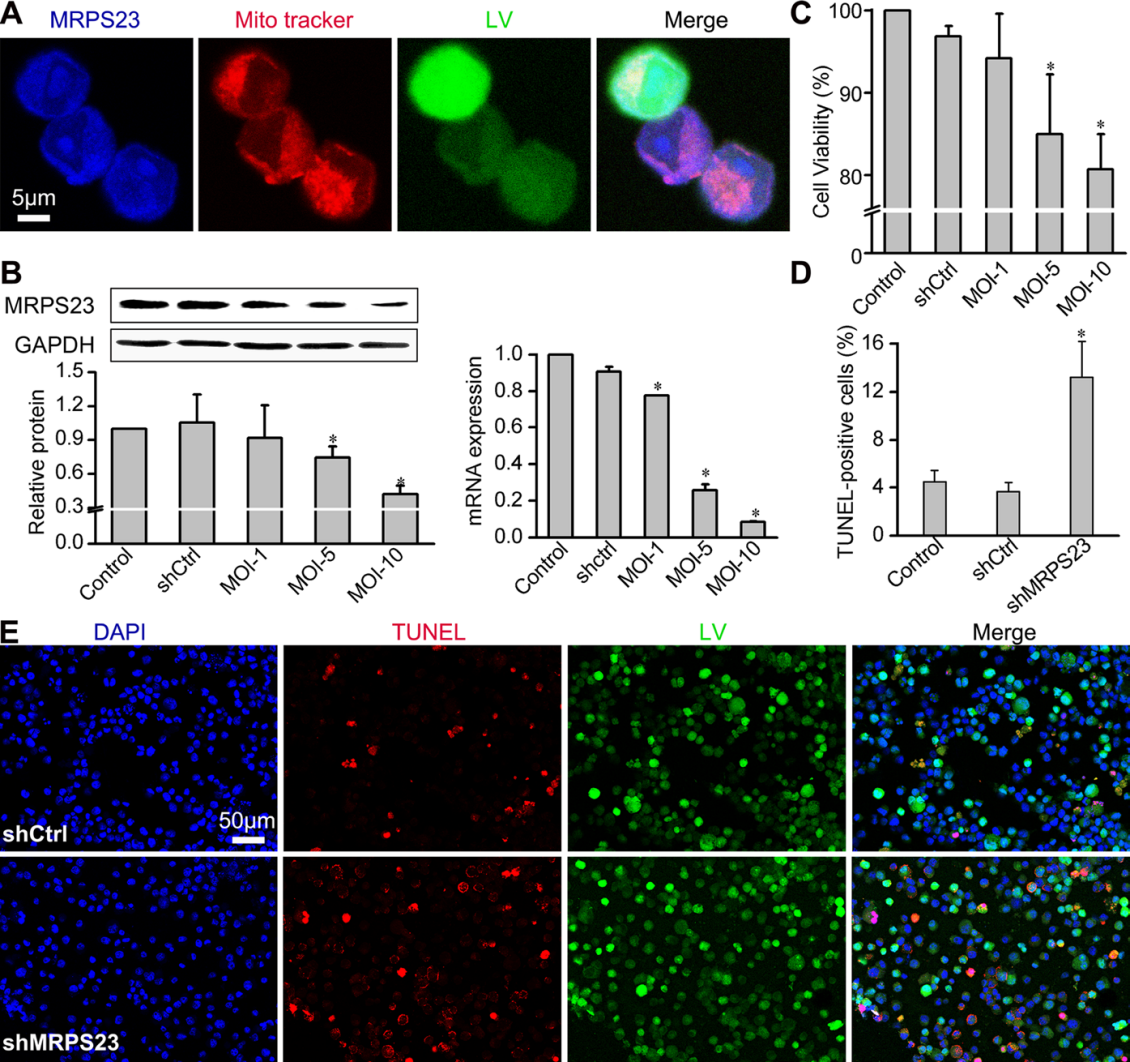
Downregulation of MRPS23 reduces Walker256 carcinoma cell viability and induces apoptosis (**A**) Representative images of subcellular localization of MRPS23 by co-staining with 100 nM MitoTracker Red (pseudo-coloured red) and anti-MRPS23 (pseudo-coloured blue). Walker 256 cancer cells was infected with LV-shMRPS23. (**B**) Efficiency of LV-shRNA interference was detected by real-time PCR and western blotting. (**C**) Walker256 carcinoma cell viability was detected by MTS assay. (**D**) Quantification of the apoptotic cells was presented as the percentage of apoptotic cells. (*p < 0.05 vs. control condition). (**E**) Confocal imaging of LV (green fluorescent), TUNEL (red fluorescent) and DAPI (blue fluorescent) staining in Walker256 cells 48 h post transfection.

To validate the efficiency of shMRPS23 knockdown and obtain an appropriate multiplicity of infection (MOI), we examined the expression of MRPS23 in Walker256 cells that were successfully infected with LV-shMRPS23 vectors at a MOI of 1, 5 and 10 respectively. The subcellular fractionation experiments using confocal immunofluorescent microscopy showed that MRPS23 (blue) was localized in the mitochondria (red) and nuclear in Walker256 cells (Figure 1A). Compared with the LV-shCtrl and PBS control groups, LV-shMRPS23 significantly inhibited MRPS23 mRNA and protein expression in cell culture (*p* < 0.05) (Figure 1B) at a MOI of 5. MTS experiments showed that knockdown of MRPS23 inhibited breast cancer cell proliferation *in vitro* compared to controls (Figure 1C). The infection rate was about 80–90%, but TUNEL-positive cells were more frequently observed 48 h post transfection with shMRPS23 (Figure 1E). Quantitative analyses revealed that the cellular apoptosis rate was about 4% in Control, shCtrl and 13.2% in shMRPS23 groups (*p* < 0.05) (Figure 1D).

### shMRPS23 inhibited breast cancer proliferation and angiogenesis *in vivo*

**Figure 2:**
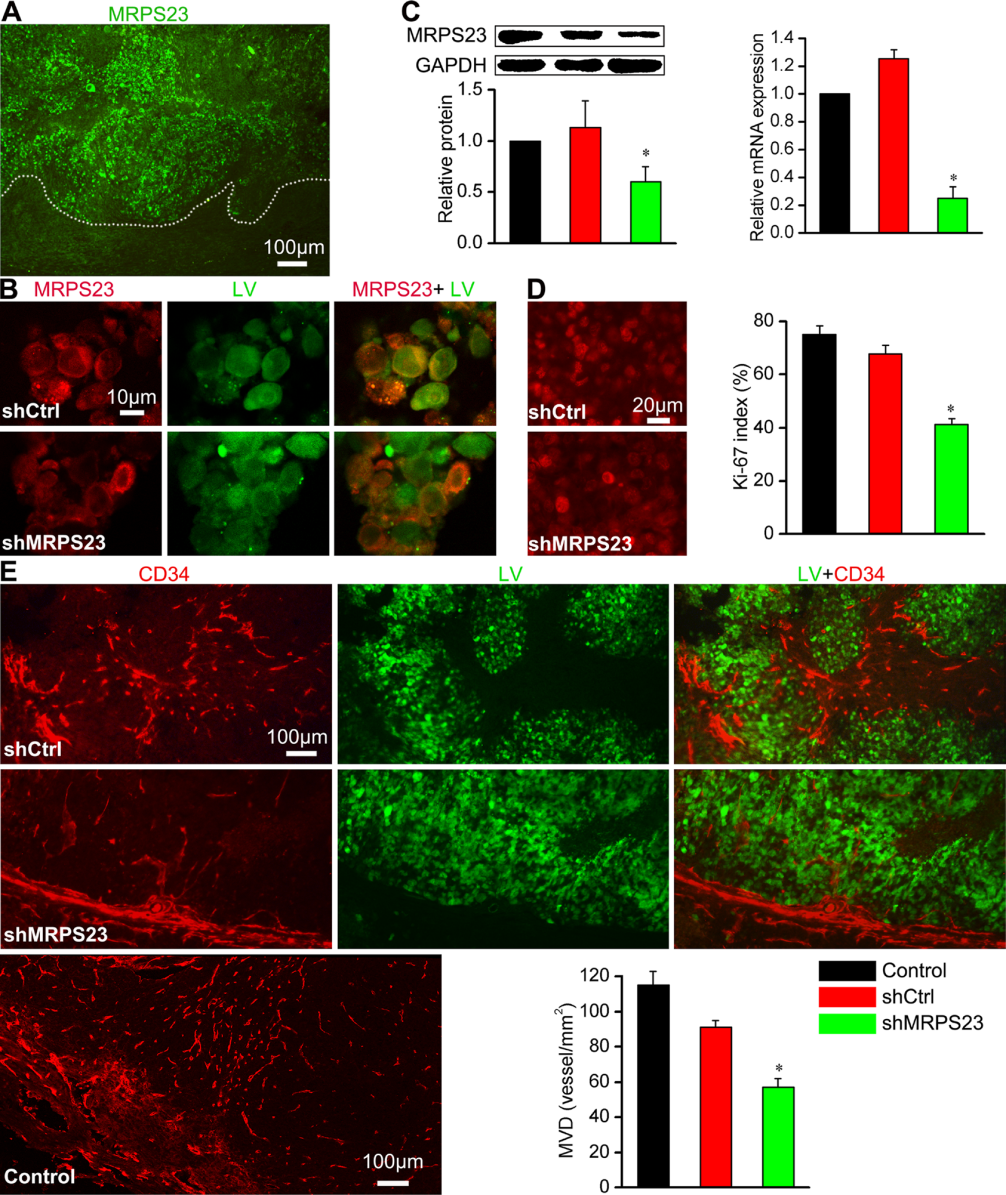
shMRPS23 inhibits cancer cell proliferation and angiogenesis *in vivo* (**A**) MRPS23 strongly expressed in tumour tissues, the white dotted line is drawn to distinguish viable tumour from tumour necrosis. (**B**) Localization of MRPS23 (red) in shCtrl or shMRPS23 treated tumour. (**C**) MRPS23 was silenced in tumour cells by LV-shRNA *in vivo*. (**D**) Representative image of immunohistochemical staining of proliferation marker Ki-67 expression in each group and the percentage of Ki-67-positive cells (overlap between Ki67 and virus) in each field. (**E**) Characterization and images of vascular network by staining for CD34 and quantification of MVD (vessel/mm^2^) in each group.

To further study the biological function of MRPS23 in breast cancer progression *in vivo*, we first investigated the expression of MRPS23 in breast tumour tissues. Immunohistochemistry revealed that MRPS23 is strongly expressed in tumour tissues compared with adjacent non-tumour tissue (Figure 2A). The colocalization of MRPS23 with LV depicted the inhibited expression of MRPS23 in shMRPS23-treated tumour (Figure 2B). Knockdown of MRPS23 also inhibited the protein and mRNA expression of MRPS23 in shMRPS23-treated tumours, compared with Control and shCtrl groups (*p* < 0.05) (Figure 2C). To illustrate whether the decreased MRPS23 contributes to breast cancer cell proliferation and angiogenesis, we performed ki-67 and CD34 stain, respectively. The proliferation index is typically 72% −78% for Control group, 64%–70% for shCtrl tumour, and 38%–40% for shMRPS23 tumour (Figure 2D). Moreover, CD34 was concentrated at the tumour- adjacent mucosa sites (Figure 2E). There was a significant difference in mean MVD (*P* < 0.05) between shMRPS23-related tumour and control tumour. Taken together, these data suggested a suppressive role of shMRPS23 in breast cancer proliferation and angiogenesis *in vivo*.

### MRPS23 knockdown suppressed breast cancer progression by upregulating p53 and p21^WAF1/CIP1^

**Figure 3:**
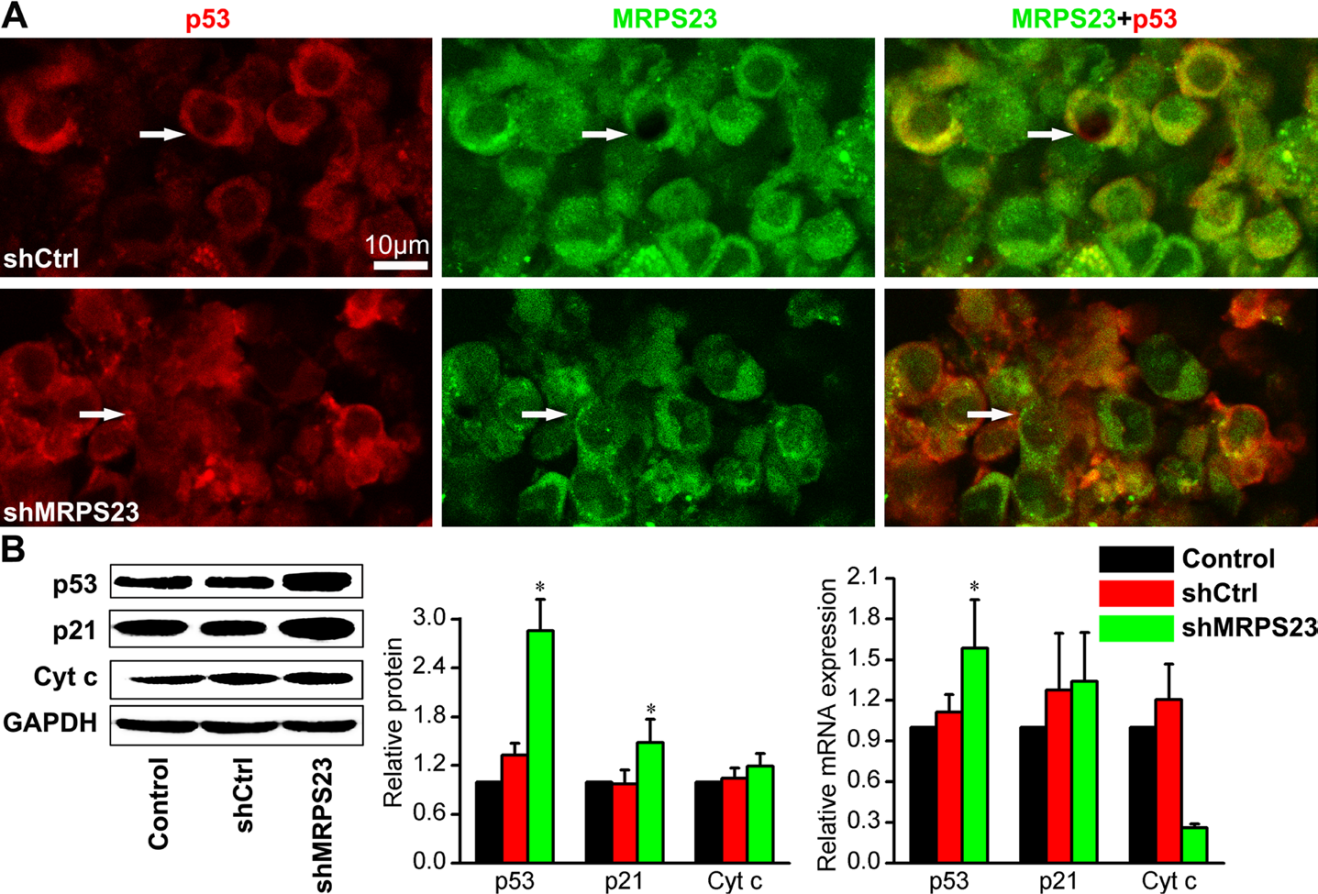
MRPS23 knockdown activates p53 and p21^WAF1/CIP1^ (**A**) Immunofluorescence staining of tumor sections showing that p53 (pseudo-coloured red) expression increases while MRPS23 (pseudo-coloured green) expression decreases after LV-shMRPS23 transfection. (**B**) Western blot and qPCR analysis of p53 and p21^WAF1/CIP1^ (p21) in the tumours of each group, showing the molecular effects induced after shMRPS23 treatment. (*p < 0.05 vs. control condition).

Given the critical role of MRPs on differential regulation of p53, p21^WAF1/CIP1^ and Cyt c levels in tumourigenesis, we wondered whether MRPS23 was associated with this signalling pathway. As expected, shMRPS23 colocalized with p53 (Figure 3A), and the mRNA and protein expression levels of p53 dramatically increased by 1.5–4 fold in shMRPS23-treated tumours (*p* < 0.05) (Figure 3B). Concomitant with the induction of p53, treatment with shMRPS23 significantly increased the level of p21^WAF1/CIP1^ protein (*p* < 0.05) (Figure 3B). However, this was not accompanied by an increase in p21^WAF1/CIP1^ mRNA. In addition, no differences were found in Cyt C mRNA expression. Knockdown of MRPS23 did not trigger the release of Cyt C (Figure 3B) in control and shMRPS23-treated conditions. These observations suggest that depletion of MRPS23 induced the expression of p53 and p21^WAF1/CIP1^ protein.

### shMRPS23 reduced the relative tumour metabolism and inhibited tumour metastasis as detected by ^18^F-FDG microPET imaging

**Figure 4:**
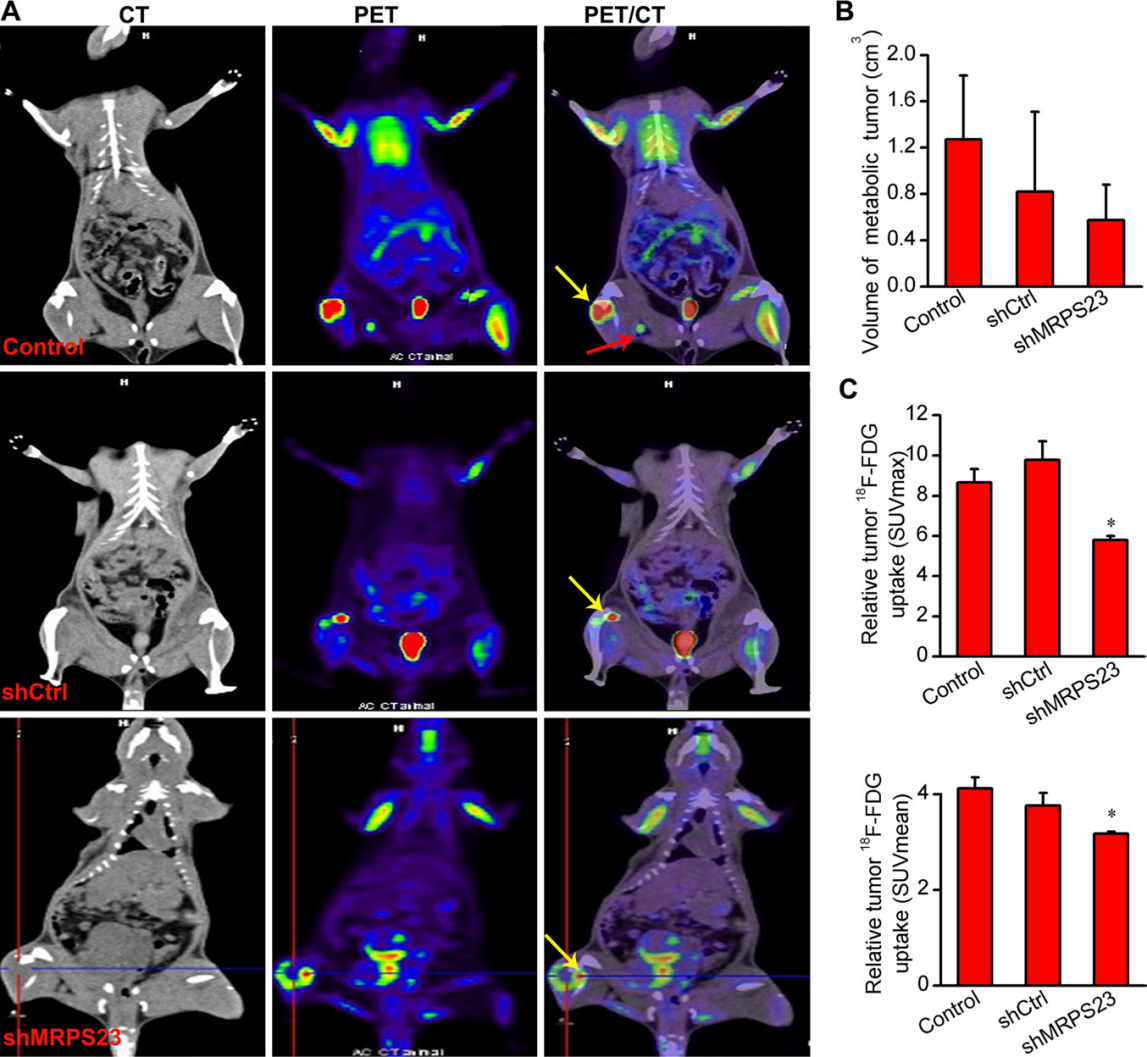
^18^F-FDG PET analysis of tumour metabolism and metabolic tumour volume (**A**) Co-registered PET images and CT images from SD rats with ^18^F- FDG injected after four weeks treatment with shMRPS23, (*n* = 4); yellow arrow depicts SUVmax sites and liquefactive necrosis (Criss Cross), red arrow indicates lymph nodes metastasis. (**B**, **C**) Quantification of metabolic volume and FDG uptake of tumours in the three experimental groups. The tumor uptake of FDG was markedly reduced in the shMRPS23-treated rat compared to the control groups (*p < 0.05 vs. control condition).

To investigate the effect of shMRPS23 treatment in our breast cancer model, we performed ^18^F-FDG PET/CT imaging. Representative images are shown in Figure 4A, VOIs were drawn to first determine metabolic volume of lesion (Figure 4B) and then estimate focal FDG uptake using SUVmax and SUVmean (Figure 4C). We observed apparent bone destruction in the right hindleg compared to the contralateral side. Importantly, increased FDG uptake in the centre of tumour focus was observed in the non-treated rats, whereas liquefactive necrosis was seen in the shMRPS23-treated rat (yellow arrow). The metabolic volume of VOIs steadily decreased in shMRPS23, yet not significantly. Notably, there was no significant difference between the Control and shCtrl group in terms of SUV elevations. Nevertheless, there was lower FDG uptake observed in the shMRPS23 treatment group, as indicated by a 1.5-fold decrease in SUVmax and a 1.45-fold decrease in SUVmean compared with Control group. Figure 4 depicts inguinal lymph node metastasis in rats infected with PBS (red arrow) and peritoneum metastasis in the shCtrl group. However, only one of six rats in shMRPS23 treatment group showed lymph nodes metastasis. Collectively, these data provided strong evidence that shMRPS23 reduces the relative tumour metabolism and inhibits tumour metastasis in the course of tumour progression.

### MRPS23 silencing on breast cancer metastasis via disturbing EMT

**Figure 5:**
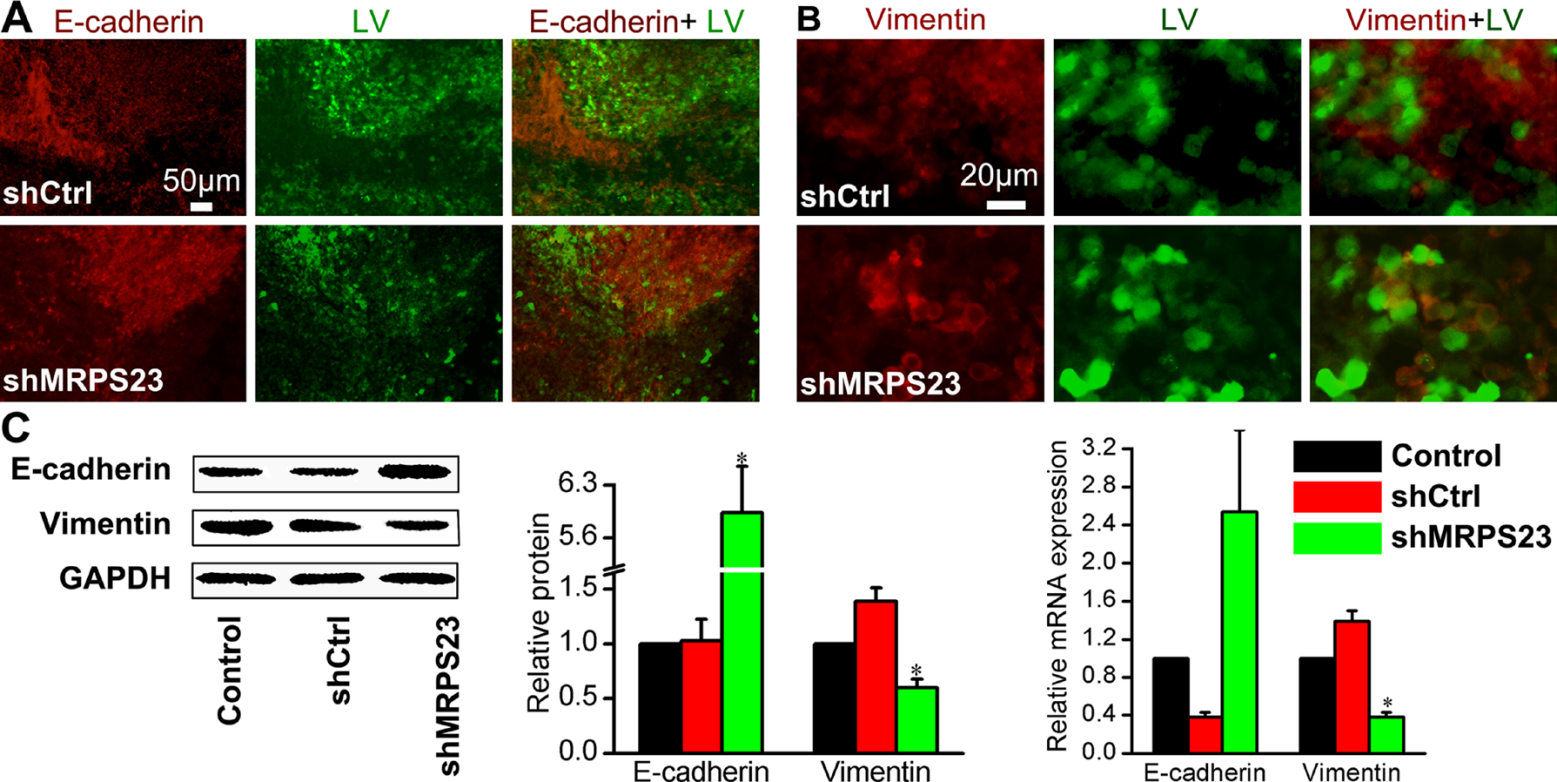
*In vivo* treatment of shMRPS23 represses breast cancer metastasis by a reversal of EMT (**A**, **B**) Walker256 tumours were immunostained with EMT markers such as E-cadherin (A) or vimentin (B) to examine the effect of shMRPS23 on EMT markers *in vivo*. (**C**) Relative protein and mRNA expression of E-cadherin and vimentin as detected by western blot and qPCR analysis in each group.

Importantly, our findings indicated that shMRPS23 impaired metastasis *in vivo* (Figure 4A). To gain additional mechanistic insight into the role of MRPS23 in the metastasis cascade, we focus on the epithelial marker E-cadherin and mesenchymal marker vimentin, which play a major role in the EMT process that contributes to tumour progression [22]. As is shown in Figure 5A, E-cadherin was localized mostly to the membrane of the leading edge in shMRPS23-treated tumours, whereas staining was diffuse to the surrounding tissue in the control groups. Conversely, vimentin expression was repressed (Figure 5B) in MRPS23 knockdown tumours compared with shCtrl-infected samples. Western analyses revealed that global levels of E-cadherin were substantially higher in the MRPS23-deficient group than in the control, whereas vimentin showed the opposite pattern (Figure 5C), as evidenced by mRNA analysis at the same time. The results suggest that MRPS23 inhibited the metastasis properties of breast cancer through influencing of EMT.

## DISCUSSION

Present genomic data sets have demonstrated that MRPS23 is upregulated in a series of cancers and it plays an essential role in breast cancer cell proliferation [5] and cervical metastasis [12]. These findings suggest that MRPS23 is tumour promoter in breast cancer. To the best of our knowledge, this is the first study to demonstrate that long-term depletion of MRPS23 complex led to repressed breast tumour growth in the xenograft model. Downregulation of MRPS23 by LV-mediated MRPS23 shRNA suppressed breast cancer metastasis shown by ^18^F-FDG microPET imaging. Moreover, shMRPS23 markedly reduced FDG uptake of breast cancer cells. In agreement with recent observations, an important finding is that the SUV value was not independent of tumour size. Our findings may supports an oncosuppressive role of MRPS23 shRNA in rat breast cancer proliferation, angiogenesis and metastasis.

However, the molecular mechanism of how breast cancer cell progression is suppresed by knockdown of MRPS23 remains to be illustrated. Mitochondrial ribosomal protein are known to be synthesized in the cytosol and then imported into mitochondria for assembly; they are responsible for translation of 13 mitochondrial mRNAs [23]. The proliferation of cultured mammalian cells can be impaired following disruption of mitochondrial function [24]. If the expression of MRPS23 was inhibited, it could disturb the mitochondrial protein synthesis, affect function of mitochondrial and impair breast cancer cell proliferation. Several studies indicate that the ribosomal proteins and MRPs may be directly involved in the process of cancer proliferation and metastasis [25, 26], which are dependent on the stimulated activity of p53 and p21^WAF1/CIP1^ [16, 27, 28]. Yoo et al. reported that the MRPL41 suppresses cell growth in association with p53, p27^Kip1^ [15] and p21^WAF1/CIP1^ [29]. Chen et al. showed that MRPS36 delays cell progression in association with p53 modification and p21^WAF1/CIP1^ expression [14]. Interestingly, we found that silencing of MRPS23 inhibited proliferation and induced apoptosis by upregulating p53 and p21^WAF1/CIP1^ protein, but not p21^WAF1/CIP1^ mRNA. The role of p53 has been shown to be to extend even beyond apoptosis, p53 can enter the mitochondrial matrix and then drive necrosis [30]. Previous studies showed that MRPs selectively act as candidate genes for mitochondrial disease [31], which is associate with the mitochondrial apoptosis [32, 33] and the release of mitochondrial Cyt c [34]. As shown in Figure 3B, shMRPS23 did not trigger the release of Cyt c. These data are consistent with the notion that the effect of shMRPS23 is associated with p53 and p21^WAF1/CIP1^ activation, but not Cyt c-mediated pathway.

Breast cancer patients are at high risk of recurrence in the form of metastatic disease [3, 35]. EMT is referred to as a key driver in the initiation of the dynamic metastatic process [36]. Consistent with a reversal of the EMT phenotype, down-regulation of MRPS23 limited the metastasis of the tumour into the surrounding lymph node, rendering the tumour smooth at its edges. During tumour progression, the tumour cells proliferation and metastasis at a distant location relies on cellular attachment to lymph or blood vessels [37]. Our data also revealed that shMRPS23 decreased the mean MVD in junctions between cancerous and non-cancerous tissues, suggesting that shMRPS23-infected cells failed to proliferate and metastasize by blocking angiogenesis. Beyond our expectation, LV-shCtrl group also showed an effect on angiogenesis compared with control group. This is consistent with the notion that lentivirus vector itself depresses angiogenesis [38].

There are several limitations to this study, including the retrospective analysis of correlation between MRPS23 and clinical prognosis in human breast cancer. Furthermore, the true potential of MRPS23 to drive tumour genesis needs to be tested in transgenic mouse models of breast cancer. Further investigations into the molecular mechanisms of MRPS23 in the progression of different breast cancer cells still remain to be elucidated. Our findings suggest that pharmaceutical intervention of shMRPS23 alone or in combination with surgery or radiation therapy might provide a promising strategy to alleviate tumour progression and metastasis.

In summary, we revealed for the first time the biological significance of MRPS23 in breast cancer. Downregulation of MRPS23 results in inhibition of proliferation and metastasis, as well as facilitating apoptosis of breast cancer cells. Additionally, shMRPS23 markedly reduced FDG uptake of the breast cancer cells and impaired angiogenic sprout *in vivo*. Thus, our findings suggested that MRPS23 might be a potential therapeutic target to alleviate breast cancer progression and metastasis.

## MATERIALS AND METHODS

### Lentivirus vector generation of anti-MRPS23 shRNA vectors

We generated short hairpin RNA (shRNA) targeting MRPS23 (GenBank accession number NM_001108289.1); the sense strand (5′-GCAGAGTCTTGGAGAAACA-3′) was cloned into the lentivirus (LV) expression construct. The vector was successfully co-transfected with LV backbone plasmid into 293T cells using lipofectamine® reagent (Invitrogen, Carlsbad, CA, USA) as reported previously [39]. LV was under the control of the U6 minimal promoter with the gene encoding GFP protein. We named the vector shMRPS23. LV construction and production were completed by Shanghai Gene Chem Co. Ltd., China. The same vector backbone but carrying a non-silencing sequence (5′-TTCTCCGAACGTGTCACGT-3′) was used as a control, and named shCtrl. The average titre of the LV was at least 5.0 × 10^8^ TU/ml.

### Quantitative real-time PCR assay

Rat mammary gland carcinoma walker 256 cells in the logarithmic growth phase were plated in 24-well plates at 1 × 10^5^ cells, and infected with PBS, LV containing shMRPS23 or LV carrying shCtrl. Total RNA was extracted from cells and tissues using the Trizol reagent (Invitrogen, Carlsbad, CA, USA) and reverse transcribed PCR using the PrimeScript™ RT Master Mix (Takara, Ohtsu, Japan). Expression levels of targeted genes were analysed by qRT-PCR on a SYBR Green qPCR Master Mix reagent system (Takara, Ohtsu, Japan). The PCR primers used are listed as follows: MRPS23: Forward 5′-TGTCAGCGGTTTGT-GGAG-3′; Reverse 5′-CAGT CTTTGCTTCTCTTACTCGTC-3′; β-actin: Forward 5′-GG AGATTACTGCCCTGGCTCCTA-3′; Reverse 5′-GACTC ATCGTACTCCTGCTTG-CTG-3′; p53: Forward 5′-GTC GGCTCCGACTATACCACTATC-3′; Reverse 5′-CTCTC TTTGCACT-CCCTGGGGG-3′; p21^WAF1/CIP1^: Forward 5′-AG TATGCCGTCGTCTGTTCG-3′; Reverse 5′-TCAAAGTT CCACCGTTCTCG-3′; Cyt c: Forward 5′-AAAGGAG GCAAGCATAAGACTG-3′; Reverse 5′- TGTTCTTGTT GGCATCTGTGT-3′; E-cadherin: Forward 5′-TCTCT TGTCCCTTCCA-CAGC-3′; Reverse 5′-CTCCAGACC CACACCAAAGT-3′; Vimentin: Forward 5′-GGATTT CTCTG-CCTCTTCCA-3′; Reverse 5′-CACCTGTCCGT CTCTGGTTT-3′.

### Western blot analysis

Cell and tissue was homogenized in RIPA lysis buffer (Promega, Madison, US) on ice for 30 min. The concentration of the total protein was measured by the BCA assay (Beyotime, Beijing, China). Western blot analyses were performed as previous study. The antibodies used in this study were as follows: anti-MRPS23 (1:1500; Santa Cruz Biotechnology, Santa Cruz, CA), anti-E-cadherin and anti-Cyt c (1:2000–5000; Abcam, Cambridge, UK), anti-GAPDH, anti-p53, anti-p21^WAF1/CIP1^, and anti-vimentin (all 1:1000; Cell Signaling Technology).

### Cell proliferation and TUNEL stain assay

Cell viability was measured by Cell Titer 96 AQueous One Solution Cell Assay (MTS, Promega, Madison, US) at 48 h post transfection. The cells were plated in triplicate at the same initial density. The terminal deoxynucleotidyl transferase dUTP nick-end labelling (TUNEL, KeyGEN Biotec, Nanjing, China) assay was performed to assess the cellular apoptosis 48h post LV shRNA infection. Confocal laser scanning microscopy images of Walker 256 carcinoma cells, triple labelled with EGFP (green pseudocolour), TRITC-TUNEL (red pseudocolour) and DAPI (blue pseudocolor) was used to analyze apoptotic index as previously described [40].

### Tumour and animal model

For *in vivo* experiments, ascites Walker 256 tumour cells were prepared and suspended in 10 μl isotonic saline containing 10^5^ cells. For implantation, in brief, fifteen Specified pathogen free female Sprague Dawley (SD) rats (200–250 g) were obtained and housed in the Experimental Animal Center of Hubei University of Medicine (Shiyan, China). For implantation, in brief, animals were anesthetized with isoflurane (3% induction, 2% maintenance), and tumour cells respectively infected with LV expressing shRNA control (shCtrl), LV expressing shMRPS23 (shMRPS23) or PBS (Control) were injected into the bone cavity as previous described [41, 42]. After surgery, rats were placed on a warm pad for recovery, randomly divided into three groups (*n* = 5), and allowed access to food and water ad libitum. All animal experimental protocols were approved by the Institutional Animal Care in the Hubei University of Medicine Taihe Hospital, and the experiments were carried out in accordance with the approved guidelines.

### *In vivo* PET/CT imaging

To assess the metabolism of tumour, four rats of each group were prepared for PET imaging 4 weeks after tumour cell injection. The animals were warmed and anesthetized throughout the entire imaging procedure, and then injected with 0.1 mCi ^18^F-FDG via a tail vein. After 1 h of unconscious uptake, images were acquired on a micro-PET/CT scanner (Siemens Inc., Erlangen, Germany) in the previous study [43]. The quantitative evaluation was performed by drawing 3D region of interests (ROIs). Maximum standardized uptake values (SUVmax) normalized for body weight and total radioactivity and metabolic tumour volume were calculated. After the PET scanning, the animals were sacrificed with an overdose of isoflurane on the next day and tumour tissues were retrieved for the following analysis.

### Immunofluorescence and immunohistochemistry assay

Walker256 cells were cultured and infected with the indicated LV for 48 h, then stained with 50 μM MitoTracker Deep Red FM (Invitrogen, Carlsbad, CA) at 37°C for 30 min. This was followed by incubation with MRPS23 primary antibody and with Donkey anti-goat Alexa Fluro 405 (1:400; Abcam, Cambridge, UK) as previously reported [44]. In fresh-frozen tumour sections, tumours were fixed in 4% paraformaldehyde, embedded with OCT, frozen at −20°C, cut into slices with 20 μm thickness. Protein antigens were detected by staining with the indicated primary antibodies overnight at 4°C, followed by incubation with a secondary antibody at 25°C for 30 min. Donkey anti-rabbit Alexa Fluor-568 and Donkey anti-mouse Alexa Fluor-405 antibodies (1:400, Abcam, Cambridge, UK) were used as secondary antibodies. Immunofluorescent labelling tissues were observed under a Leica fluorescent inverted microscope. The microvessel density (MVD) was assessed by CD34 Ab staining with the method described in the previous study [20, 45].

### Statistical data analyses

The results were expressed as means ± SD. All statistical analysis was performed by SPSS 17.0 software. One-way ANOVA was used to compare the differences between groups. *p < 0.05 was considered statistically significant.
